# Differential Effects of Lovastatin on Cisplatin Responses in Normal Human Mesothelial Cells versus Cancer Cells: Implication for Therapy

**DOI:** 10.1371/journal.pone.0045354

**Published:** 2012-09-17

**Authors:** Yandong Shi, Emanuela Felley-Bosco, Thomas M. Marti, Rolf A. Stahel

**Affiliations:** Laboratory of Molecular Oncology, University Hospital of Zürich, University of Zürich, Zürich, Switzerland; King Faisal Specialist Hospital & Research Center, Saudi Arabia

## Abstract

The cancer killing efficacy of standard chemotherapeutic agents such as cisplatin (CDDP) is limited by their side effects to normal tissues. Therefore, research efforts optimizing the safety and efficacy of those agents are clinically relevant. We did screen for agents that specifically protect normal human mesothelial cells against CDDP without reducing the cancer cell killing efficacy. Lovastatin was identified from the screen. Lovastatin at a pharmacologically relevant concentration strongly arrested the proliferation of normal cells, whereas cancer cells were less affected. CDDP-induced DNA damage response was not activated and normal cells showed enhanced tolerance to CDDP when normal cells were treated with the combination of CDDP and lovastatin. We demonstrate that interfering with protein geranylgeranylation is involved in the lovastatin-mediated CDDP protective effect in normal cells. In contrast to normal cells, in cancer cells lovastatin did not change the CDDP-induced response, and cancer cells were not protected by lovastatin. Furthermore, lovastatin at the pharmacological relevant concentration *per se* induced DNA damage, oxidative stress and autophagy in cancer cells but not in normal mesothelial cells. Therefore, our data suggest that lovastatin has a potential to improve the therapeutic index of cisplatin-based therapy.

## Introduction

Cisplatin (CDDP) is a standard chemotherapeutic agent for the treatment of various solid tumors. However, side effects to normal tissues result in limited tolerance in patients [1]. Thus, therapeutic strategies circumventing the toxic side effects of CDDP would be welcome and might improve the therapeutic outcome.

Loss or weakened cell cycle checkpoints are among the most universal alterations in cancer cells, resulting in reduced sensitivity to proliferation-inhibitory signaling that normally initiate a variety of responses including proliferation arrest, activation of self-protection mechanisms against stresses, differentiation, or cell death [2]. Under certain proliferation-limiting conditions, normal cells arrest their replication and thereby may be protected from the toxicity of proliferation-dependent agents, e.g. the DNA-damaging agents. However, cancer cells, due to their reduced sensitivity to proliferation-inhibitory signaling, cannot properly arrest their cell cycle and are therefore selectively killed under these conditions [3]–[6].

Our aim was to find agents which could protect normal cells against cisplatin cytotoxicity and at the same time allow killing cancer cells. Therefore, we set up a two-step screen: first, we screened a series of compounds for differential effects on the proliferation of normal mesothelial *versus* mesothelioma cells; second, combined actions of CDDP with those differentiating agents were tested in normal cells to identify agents which make normal cells tolerant to CDDP cytotoxicity while allowing cancer cells killing.

Lovastatin was identified from our screen. As a cholesterol-lowering drug, lovastatin acts by inhibiting HMG-CoA reductase, the rate-limiting enzyme of the cholesterol biosynthesis pathway [7]. Blocking the cholesterol synthesis pathway also results in the depletion of isoprenoid moieties thereby interfering with protein isoprenylation, which is a crucial regulatory step in many biological procedures [7]. Therefore, beyond the cholesterol-lowering function, lovastatin also performs pleiotropic biological roles in the control of cell proliferation, apoptosis, survival, differentiation, migration and cellular vesicle trafficking [7]–[9]. Our data show that lovastatin has a potential to increase the therapeutic index of CDDP.

**Figure 1:**
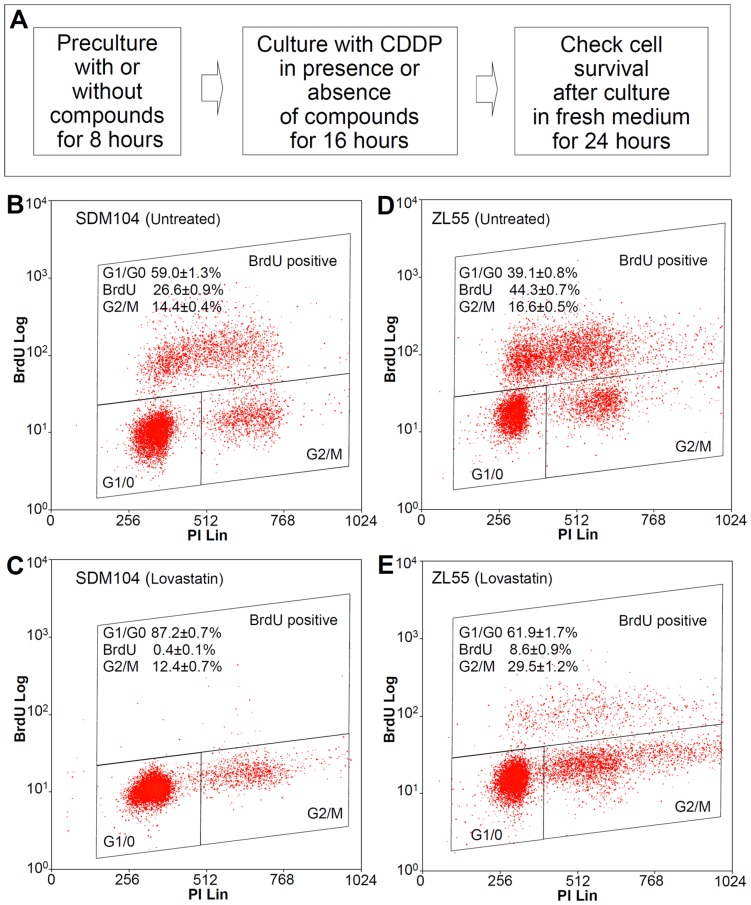
Lovastatin differentially affected the cell proliferation of normal *versus* cancer cells *in vitro*. The experimental protocol for the investigation of agents protecting normal cells against CDDP cytotoxicity is shown in (**A**). Cell cycle profiles of normal SDM104 (**B**) and (**C**), and ZL55 cancer cells (**D**) and (**E**) of control without treatment (**B**) and (**D**), or 24 hours treatment with 2 µM lovastatin (**C**) and (**E**) are shown (n = 3). Cells were exposed to 10 µM BrdU for one hour before harvesting for FACS analysis.

## Materials and Methods

### Cell Cultures and Reagents

Normal human mesothelial primary cells and cancer cells were cultured in M199 (Invitrogen)/MCDB105 (Sigma) (1∶1) mixed medium supplemented with 15% FCS, 10 ng/ml EGF, and 0.4 µg/ml hydrocortisone as described [10]. The human mesothelioma cell line ZL55 [11] and the primary normal cell culture SDM104 [12] were generated in our laboratory. The breast cancer cell line MCF-7 [13] and the human lung adenocarcinoma cell line A549 [14] were purchased from American Type Culture Corporation (Manassas, VA). The primary normal cell culture LP9 [15] was from Dr. James Rheinwald’s laboratory. The primary normal cell culture SDM85 was established from a normal pleural tissue received from a patient undergoing cancer unrelated thoracic surgery. The study was approved by the Zürich University Hospital ethic committee and a written informed consent was obtained from the patient. All cell lines used in this study were authenticated by fingerprinting (Microsynth, Balgach, Switzerland). Lovastatin (Alexis Biochemicals) was converted into the active acid form as described [16] and used at a concentration of 2 µM in most experiments. Cisplatin (0.5 mg/ml saline solution) was purchased from Ebewe Pharma; mevalonate, GGPP and FTI-277 from Sigma; GGTI-298 from Calbiochem. Anti-ATM-Ser1981 (Epitomics), anti-ATM (2C1) (Gene Tex), anti-γ-H2AX (Ser139) (Millipore), anti-LC3B (Abcam), anti-phospho-p53 (Ser15) (Cell Signaling Technology), anti-p53, anti-β-Actin, anti-Heme Oxygenase 1 (HO 1), anti-p21, anti-H-Ras, anti-Rap1a and anti-vinculin (Santa Cruz) were used according to the product instructions. For western blot detection of ATM, protein extracts were run in 5% SDS-polyacrylamide gel while for the rest 13.5% SDS-polyacrylamide gel was used.

**Table 1:** Different effects of agents on the cell cycle distribution of normal versus cancer cells.

Agents	ZL55	SDM104
Hsp90 inhibitor 17-AAG (0.25µM)	G1 and G2/M arrest	G2/M arrest, still S phase cells
EGFR inhibitor AG1478 (20µM)	G1 arrest	No response
Akt Inhibitor II (20µM)	G1 arrest	No response
PKC inhibitor GF109203X (10µM)	G1 arrest	No response
Lovastatin (2µM)	G1 delay, still S phase cells	G1 and G2/M arrest
Nocodazole (50nM)	G2 arrest and cell death	G1 and G2/M arrest and cell death
Pemetrexed (10nM)	G1 and S arrest	No response
Kinase inhibitor UCN-01 (25nM)	G1 arrest	No response

**Figure 2:**
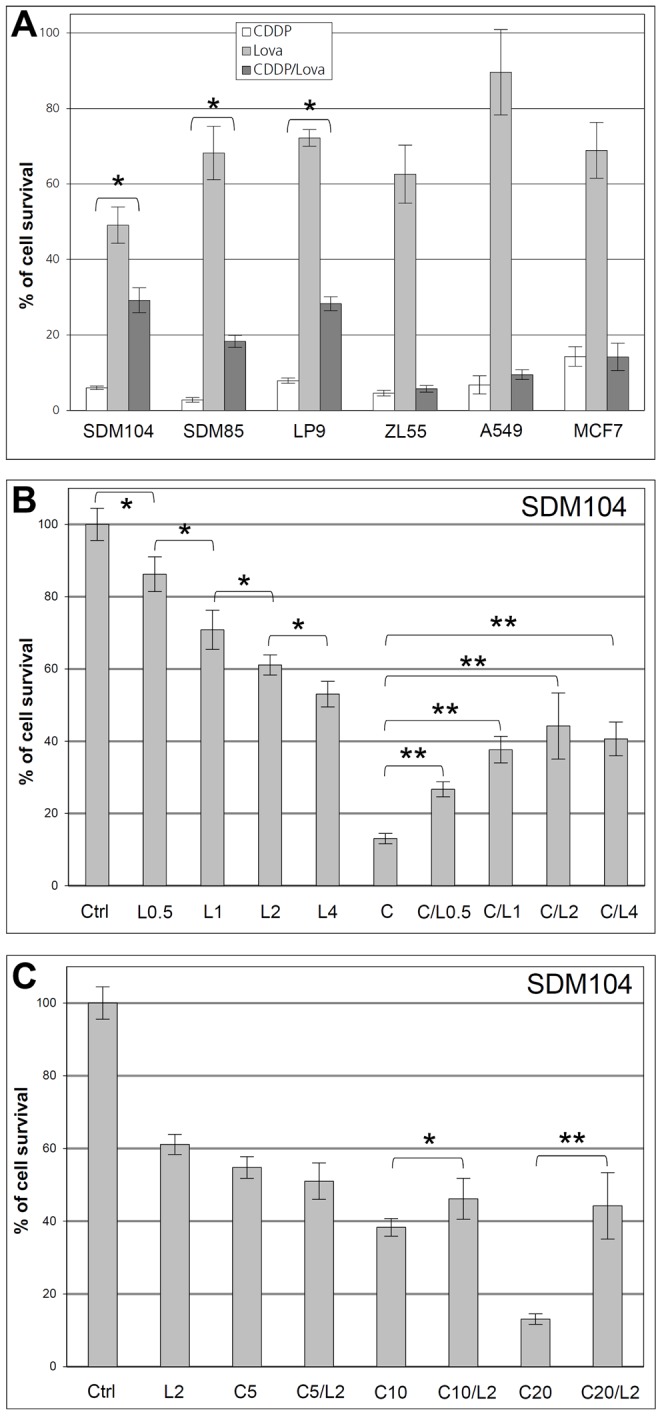
Lovastatin specifically protecting normal but not cancer cells from CDDP toxicity *in vitro*. Results of MTT assays with primarily cultured normal cells (SDM104, SDM85 and LP9) and cancer cells (ZL55, A549 and MCF-7) after treatments with CDDP alone, lovastatin (2 µM) alone, or both together are shown in (**A**) (n = 6; * for P<3.0×10^−5^). In (**B**), MTT assays were performed after SDM104 cells were treated with different concentrations of lovastatin alone, or lovastatin together with 20 µM CDDP (n = 6; * for P<0.002; ** for P<3.0×10^−5^); L0.5, L1, L2 and L4 stand for 0.5 µM, 1 µM, 2 µM and 4µM lovastatin, respectively; and C for CDDP. In (**C**), MTT assays were performed after SDM104 cells were treated with 2 µM lovastatin alone, CDDP of different concentrations alone, or both together (n = 6; * for P<0.02; ** for P<1.0×10^−5^); C5, C10, and C20 stand for 5 µM, 10 µM and 20 µM CDDP, respectively.

**Figure 3:**
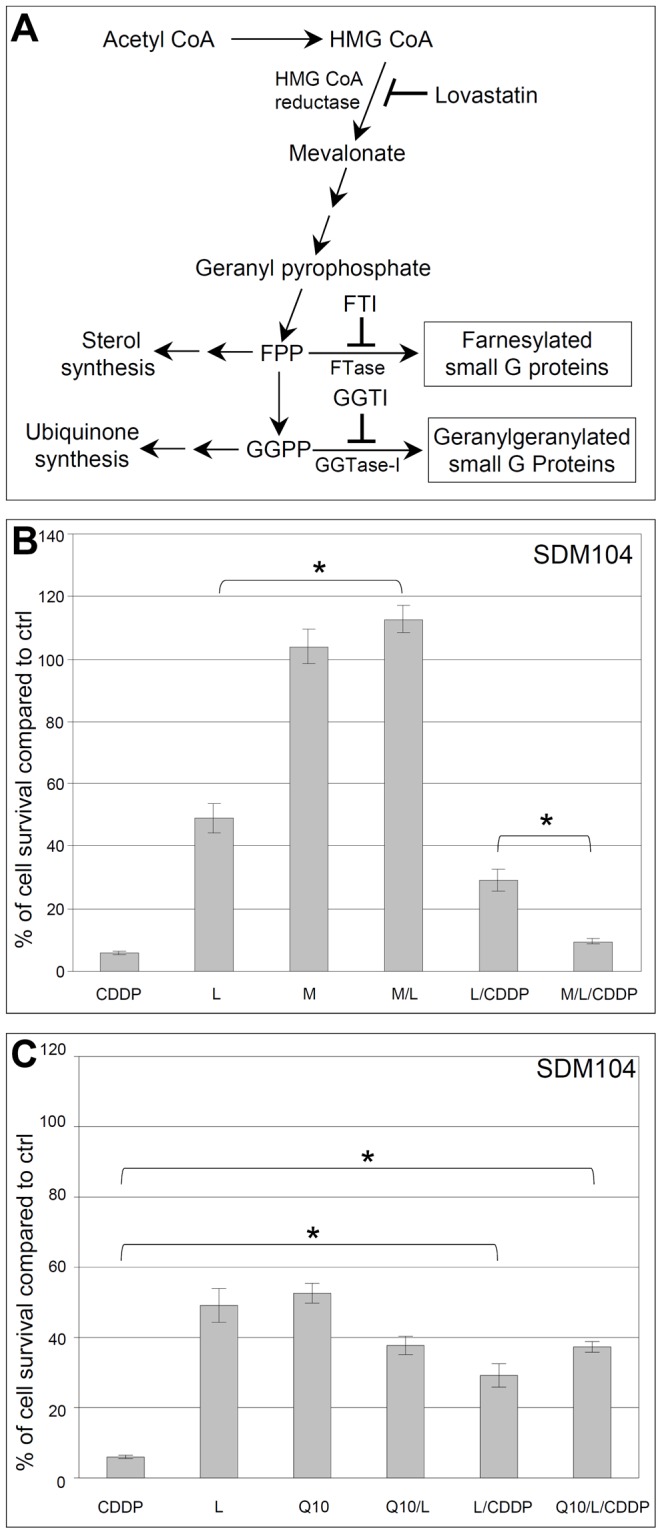
Blocking the cholesterol biosynthetic pathway is involved in the lovastatin-mediated protection of normal cells. A scheme for the cholesterol biosynthetic pathway is shown in (**A**). In (**B**) and (**C**), 20 µM CDDP were added after 8 hours pre-incubation with 2 µM lovastatin in the presence or absence of 200 µM mevalonate (B) or 100 µM Q10 (C), then cells were cultured for another 16 hours in the presence of CDDP and the other agents together (n = 6; * for P<7.0×10^−7^). MTT assays were performed after the treatments ended and cells were cultured again in fresh medium for 24 hours. In (**B**) and (**C**), “L” stands for lovastatin, “M” for mevalonate.

### Cell Proliferation and Cell Cycle Analysis

MTT cell proliferation assay was performed as described [17]. For the compound screen, the analysis of the cell cycle distribution was done as following: cells were harvested after 24-hour treatment with different agents, washed with PBS and fixed in 70% ethanol overnight at 4°C. After propidium iodide (PI) (Sigma) staining, flow cytometry (FACS) analysis was performed with FACSCalibur (FACScan, BD Biosciences) and data was analyzed with ModFit LT 3.2.1 software. For the detailed FACS analysis of lovastatin-treated cells, cells were exposed to 10 µM BrdU for one hour before harvesting. Cells were harvested after 24 hour lovastatin-treatment and fixed in 70% ethanol. After anti-BrdU antibody (BD Biosciences)/Secondary Alexa488-conjugated goat-anti-mouse (Invitrogen) and PI staining, FACS analysis was performed with FACSCalibur and data was analyzed with Summit v4.3 software. The statistical significance was performed with two-tailed t-Test.

**Figure 4:**
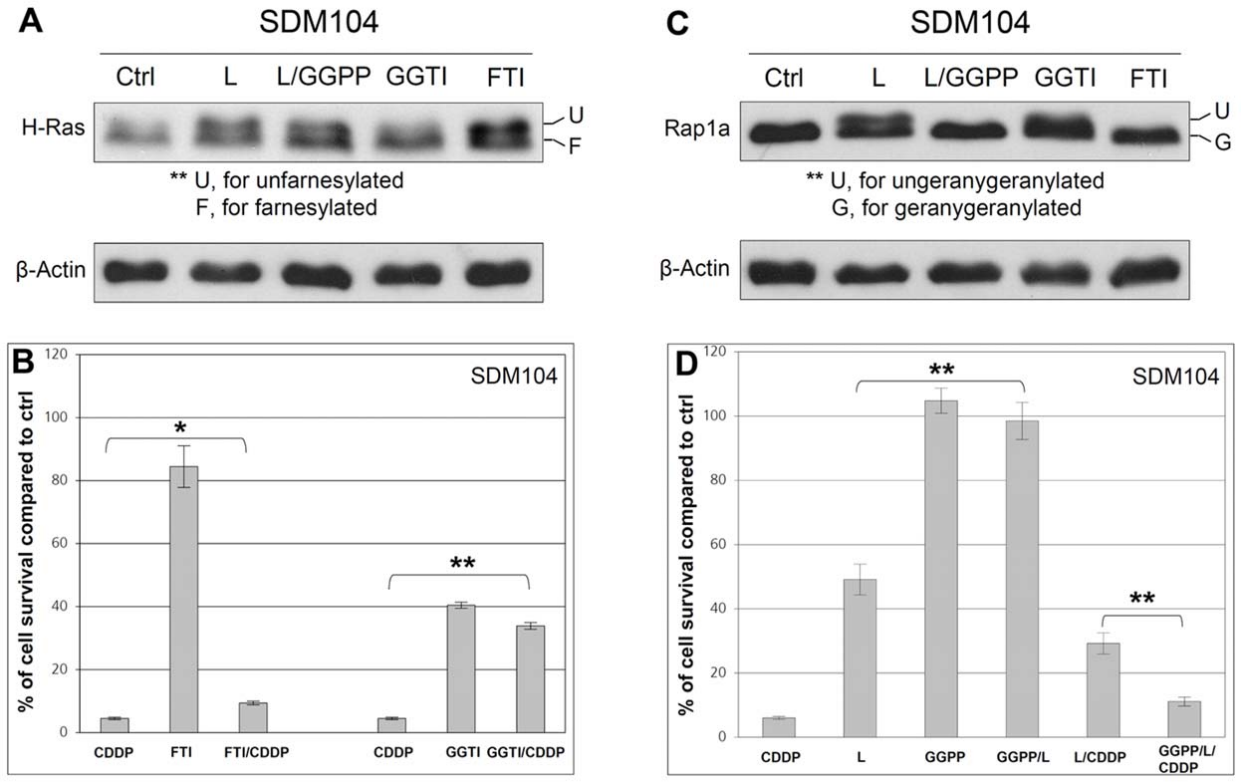
Protection of normal cells from cisplatin toxicity is through interference of protein geranylgeranylation. Results of Western blot with anti-H-Ras antibody (**A**) and anti-Rap1a antibody (**C**) are shown. Protein extracts were made right after normal SDM104 cells were treated with different compounds for 16 hours. β-Actin used as loading control for Western. In (**B**), 20 µM CDDP were added after 8 hours pre-incubation with 10 µM GGTI-298 (GGTI) or 10 µM FTI-277 (FTI), and then cells were cultured for another 16 hours in the presence of CDDP and the inhibitors together (n = 6; * for P<3.0×10^−4^; ** for P<2.0×10^−6^). In (**D**), 20 µM CDDP were added after 8 hours pre-incubation with 2 µM lovastatin in the presence or absence of 10 µM GGPP, and then cells were cultured for another 16 hours in the presence of CDDP and the compounds together (n = 6; ** for P<2.0×10^−6^). In (**B**) and (**D**), MTT assays were performed after the treatments ended and cells were cultured again in fresh medium for 24 hours. “L” stands for lovastatin.

**Figure 5:**
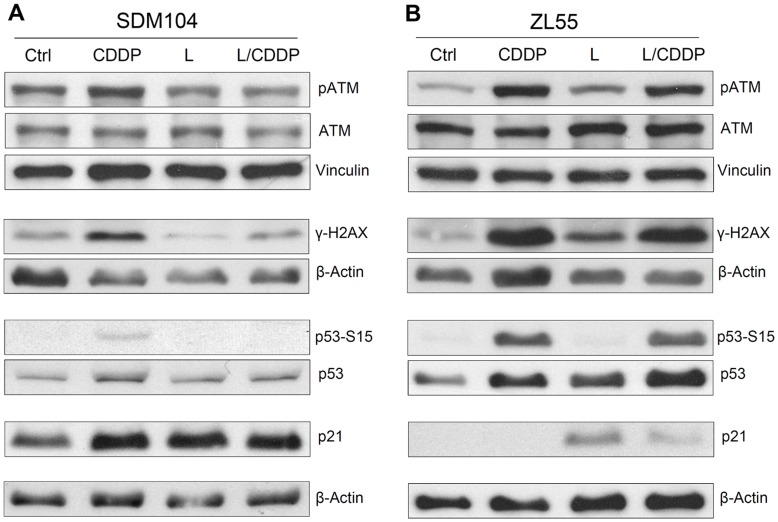
Lovastatin inhibits the activation of CDDP-induced DNA damage responses in normal cells but induces DNA damage responses in cancer cells. Western blot analysis of components in DNA-damage response in SDM104 normal cells (A) and cancer cells (B) after treatment with CDDP in the presence or absence of lovastatin are shown. Vinculin and β-actin were used as loading control. For CDDP treatment, cells were cultured in the presence of 8 µM CDDP for 16 hours; for lovastatin, cells were cultured in the presence of 2 µM lovastatin for 24 hours; for the combination of CDDP and lovastatin, 8 µM CDDP were added 8 hours after 2 µM lovastatin pre-incubation, then cells were cultured for another 16 hours in the presence of CDDP and lovastatin together.

## Results

### Lovastatin Differentially Affected the Cell Proliferation of Normal *versus* Cancer Cells and Specifically Protected Normal Cells from CDDP Cytotoxicity *in vitro*

The screen for agents which could protect normal cells against cisplatin-induced cytotoxicity was performed in two steps. In the first step, we studied the literature and selected candidate agents from which we found indications that they might differentially affect proliferation of normal *versus* cancer cells. Cell cycle profiles of normal human mesothelial SDM104 cells and human mesothelioma ZL55 cells were analyzed 24 hours after exposure to commercially available agents known to interfere with cell proliferation. Some of the agents showing different effects on the cell cycle distribution in normal *versus* cancer cells are listed (Table 1). In the second step, we tested which of the selected agents protected normal cells from CDDP (Figure 1A). Lovastatin, an FDA-approved cholesterol-lowering drug, was identified in the second step.

**Figure 6:**
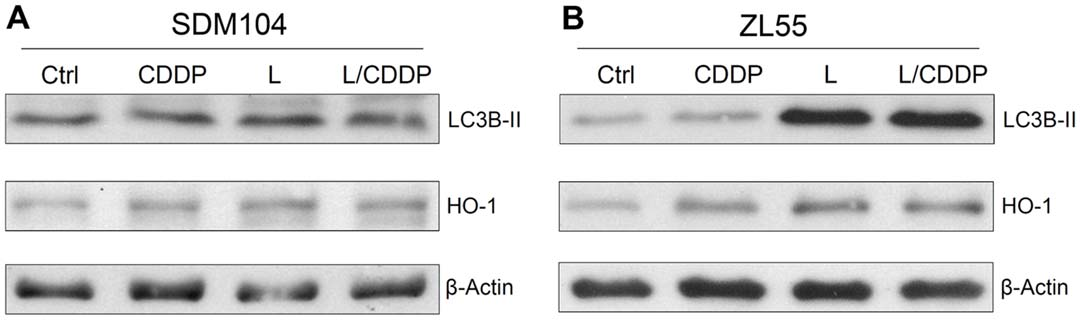
Lovastatin induces oxidative stress and autophagy in cancer but not normal cells. Western blot analysis of autophagy marker LC3B-II and oxidative stress marker HO-1 in normal (SDM104) cells (**A**) and cancer (ZL55) cells (**B**) after treatment with CDDP in the presence or absence of lovastatin are shown. β-Actin were used as loading control. For CDDP treatment, cells were cultured in the presence of 8 µM CDDP for 16 hours; for lovastatin, cells were cultured in the presence of 2µM lovastatin for 24 hours; for the combination of CDDP and lovastatin, 8 µM CDDP were added 8 hours after 2 µM lovastatin pre-incubation, then cells were cultured for another 16 hours in the presence of CDDP and lovastatin together.

Lovastatin at the pharmacological relevant concentration [18] of 2 µM in normal cells reduced more than 98% of the S-phase cells and about 14% of the G2/M cells while there was about 47.8% increases of G0/G1 cells (Figure 1C), as compared to untreated control (Figure 1B). In contrast, in cancer cells there were still about 20% of the S-phase cells remaining and both G0/G1 cells and G2/M cells were increased 58% and 77.7%, respectively (Figure 1E) after lovastatin-treatment, compared to untreated control (Figure 1D). Thus, lovastatin showed different effects on the proliferation of normal cells *versus* cancer cells. Consistent with changes observed in cell cycle, treatment with lovastatin alone, significantly suppressed the proliferation of normal (P<1.0×10^−6^), and cancer ZL55 (P<0.001) and MCF-7 (P<0.001) cells, compared to untreated controls (Figure 2A).

The number of normal SDM104 cells after the combined treatment of CDDP and lovastatin was 3.8 times higher compared to the cell number observed after treatment with CDDP alone. This protective effect against CDDP toxicity was also observed in two additional normal primary cultures SDM85 and LP9 (Figure 2A). The lovastatin-mediated protective effect seemed specific for normal cells, since none of the tested cancer cell lines, human mesothelioma ZL55, human breast cancer MCF-7 and human lung adenocarcinoma A549, was protected (Figure 2A).

A lovastatin dose response for protective effect against CDDP cytotoxicity was performed with SDM104 cells. Lovastatin dose-dependently reduced cell growth, however, the maximal protective effect was observed already at 2 µM (Figure 2B). Thus, 2 µM lovastatin concentration was used in most of our experiments. Because lovastatin-mediated protection of normal cells was linked to cell cycle arrest, CDDP-protective effects was visible only at high CDDP concentration (10–20 µM) where reduction in cell number due to cytotoxicity was higher than lovastatin-induced cell number reduction due to proliferation arrest (Figure 2C).

### Blocking the Cholesterol Biosynthetic Pathway but not Ubiquinone Synthesis is Required for the Lovastatin-mediated Protection of Normal Cells from Cisplatin Toxicity

As a lipid lowering agent, lovastatin acts to inhibit HMG-CoA reductase, the rate-limiting enzyme of cholesterol biosynthetic pathway [7] (Figure 3A). We examined whether blocking the cholesterol biosynthetic pathway is required for the lovastatin-mediated protection of normal cells against CDDP by testing the effects of mevalonate, which is the immediate product of HMG-CoA reductase-catalyzed reaction in the cholesterol biosynthetic pathway [7] (Figure 3A). Addition of mevalonate completely reversed the inhibitory effects of lovastatin on cell proliferation. Lovastatin did not protect normal cells against CDDP in the presence of mevalonate (Figure 3B), indicating that lovastatin protects normal cells by blocking the cholesterol biosynthetic pathway. The addition of ubiquinone (coenzyme Q10) in normal cells did not change either the inhibitory effect of lovastatin on cell proliferation or the protection from CDDP (Figure 3C) indicating the interference with the ubiquinone synthesis is not involved in the observed protection of normal cells.

### Protection of Normal Cells from Cisplatin Toxicity is Mediated through Lovastatin-Induced Interference of Protein Geranylgeranylation

Lovastatin interferes with protein isoprenylation (including farnesylation and geranylgeranylation) through depleting the isoprenoid donors farnesyl pyrophosphate (FPP) and geranylgeranyl pyrophosphate (GGPP) [7] (Figure 3A, and 4A and 4C). Farnesyltransferase inhibitor (FTI-277) specifically inhibits the farnesylation of small G proteins, e.g., the farnesylation of H-Ras (Figure 4A) but not protein geranylgeranylation, e.g., the geranylgeranylation of Rap1a in normal SDM104 cells (Figure 4C). FTI-277 showed much weaker proliferation inhibitory effect on normal cells (Figure 4B) than lovastatin (Figure 3B), and its CDDP-protective effect to normal cells (Figure 4B) was also weaker than lovastatin (Figure 3B). However, the specific inhibition of geranylgeranylation by a geranylgeranyltransferase-I inhibitor (GGTI-298) (Figure 4C), which did not affect farnesylation (Figure 4A), strongly inhibited the proliferation of normal cells and protected them from cisplatin toxicity (Figure 4B) similar to lovastatin (Figure 3B). Consistent with these observations, the cell proliferation-inhibitory effects and the CDDP protective effect of lovastatin for normal cells were dramatically suppressed by the addition of GGPP (Figure 4D), which reversed the lovastatin-mediated inhibition of geranylgeranylation (Figure 4C) but not farnesylation (Figure 4A). Thus, our data demonstrates that lovastatin-mediated protection of normal cells from CDDP toxicity is mainly due to the GGPP depletion-induced inhibition of geranylgeranylation.

### Lovastatin Prevents the Activation of CDDP-induced DNA Damage Responses in Normal Cells but Induces DNA Damage Responses in Cancer Cells

To further explore the mechanism of lovastatin-mediated differential effects on normal *versus* cancer cells, the responses of normal *versus* cancer cells were examined in more details. As expected, CDDP activated the DNA damage responses [19]–[22] in normal cells: the phosphorylation of ATM, the consequent accumulation of the DNA damage marker phospho histone H2AX on Serine 139 (γ-H2AX), the phosphorylation and accumulation of p53, and the accumulation the cell cycle inhibitor p21 (Figure 5A). However, except for p21 upregulation, DNA damage response was not detected when cells were treated with lovastatin 8 hours prior and during CDDP treatment (Figure 5A), indicating that lovastatin, by arresting cell growth, suppressed the DNA damage response and protected normal cells from CDDP-induced cytotoxicity. Lovastatin-induced up-regulation of p21 was likely through a p53-independent pathway since no detectable activation of p53 was observed in normal cells treated with lovastatin alone (Figure 5A).

In cancer cells, in contrast to normal cells, the CDDP-induced DNA damage response including the activation of ATM, accumulation of γ-H2AX and p53 were not changed in the combined treatment with lovastatin (Figure 5B). Importantly, while lovastatin alone did not induce further response except p21 upregulation in normal cells (Figure 5A), it resulted in increased levels of P-ATM, p53 and γ-H2AX in cancer cells (Figure 5B) indicating that lovastatin at the pharmacological relevant concentration of 2 µM *per se* induces DNA damage in cancer cells.

### Lovastatin Induces Autophagy and Oxidative Stress in Cancer but not Normal Cells

Consistent with other studies reporting lovastatin-induced autophagy [23]–[25], we observed that lovastatin triggered the up-regulation of the autophagy marker LC3B-II [26] in cancer cells (Figure 6B). However, LC3B-II was not up-regulated in lovastatin-treated normal cells (Figure 6A). Lovastatin also induced oxidative stress indicated by the expression of heme oxygenase-1 (HO-1) [27] in cancer cells (Figure 6B), which was not detected in normal SDM104 cells either (Figure 6A). Therefore, lovastatin induces autophagy and oxidative stress in cancer but not normal cells.

## Discussion

Side effects of anticancer drugs impair patients’ life quality and affect therapeutic efficacy [28]. We identified lovastatin from a screen for agents which reduced cisplatin-induced toxicity in normal cells, while allowing cancer cells to be killed. Lovastatin not only mediated the CDDP protective effect for normal cells, but also induced DNA damage, oxidative stress and autophagy specifically in cancer cells.

The protection of normal cells against CDDP is in agreement with the previous observations that lovastatin protected human endothelial cells (HUVEC) from the toxic effects of anticancer drugs doxorubicin and etoposide and the killing of ionizing radiation *in vitro* [29], [30]. Lovastatin also reduced ionizing radiation-induced normal tissue damage and protected against anthracycline-induced cardiac toxicity *in vivo* [31], [32].

We showed that lovastatin mediated CDDP protective effect in normal cells at a concentration of 2 µM, which is in the range of the pharmacologically attainable serum concentration of lovastatin (2.3±1.27 µM) [18], meaning immediate feasibility of clinical implementation of lovastatin treatment of cancer patients receiving this drug. Similar concentration had been used for the protection of mouse CHO cells from doxorubicin, and the inhibition of geranylgeranylation was suggested to be involved in the protective effect [33]. We showed here that a similar mechanism also functions in the lovastatin-mediated protection of normal human cells from CDDP. We further confirmed that the protective effect of lovastatin is mainly due to a geranylgeranylation-dependent mechanism since GGTI but not FTI showed a similar effect as lovastatin in protecting normal human cells from CDDP toxicity.

Geranylgeranylated proteins are involved in the control of cell proliferation [9]. The reduction of this post-translational modification may be responsible for the cell proliferation arrest observed in normal cells after treatment with lovastatin. It is known that processing of CDDP-DNA-adducts in replicating cells leads to DNA damage [19], [34]. Therefore, replication-quiescent or -inhibited cells become resistant to CDDP. This may also explain why lovastatin does not protect cancer cells, where the proliferation-inhibitory effects of lovastatin may be antagonized by oncogenic mutations [35].

Recent studies have renewed the therapeutic interest for the inhibitors of cholesterol biosynthetic pathway in breast cancers with p53 mutations since they become highly reliant on this pathway [36]. In two of tested cancer cell lines (ZL55 and MCF-7) we observed a significant decrease of cell proliferation after treatment with lovastatin, indicating that even p53-proficient tumors may be sensitive to some extent to growth inhibitory effects of lovastatin therapy. In contrast to normal cells, where the effect on proliferation resulted from the inhibition of DNA replication, the lovastatin-mediated decrease of cell proliferation in cancer cells may be attributed to DNA damage-dependent G_0_/G_1_ arrest. Due to the minor decrease in S-phase cells, cancer cells were not protected by lovastatin from the cytotoxic effect of CDDP.

It is known that lovastatin suppresses proliferation, and induced oxidative stress, autophagy and apoptosis in cancer cells *in vitro* [24], [37]–[45]. In line with these observations, lovastatin inhibited tumor growth [25] and potentiated the antitumor activity of doxorubicin and CDDP *in vivo* [46], [47] and long-term use of statin is associated with lower risk of many cancers [48], [49]. Here, we further show that those lovastatin-induced detrimental effects happen specifically in cancer but not normal cells.

Therefore, our data demonstrate that lovastatin has a potential for protecting normal cells from CDDP toxicity without decreasing the sensitivity of cancer cells to CDDP-based therapy thereby improving the therapeutic index.
